# Stabilized but Rejected: The Trajectory of a Dual Disorder Patient Facing Addictive Relapse

**DOI:** 10.1192/j.eurpsy.2026.10908

**Published:** 2026-07-31

**Authors:** A. Amrandi, S. Boudour, M. T. Benatmane

**Affiliations:** EPS Barthelemy Durand, Etampes, France

## Abstract

**Introduction:**

Dual disorders, combining severe mental illness and substance use, represent a major therapeutic and social challenge. Even when psychiatric stabilization is achieved under long-acting antipsychotic treatment, addictive relapses can compromise recovery, autonomy, and social reintegration. These complex cases often lead to repeated psychiatric hospitalizations despite the absence of psychotic symptoms, highlighting the need for more integrated approaches between psychiatric and addiction services.

**Objectives:**

To describe the clinical course of a dual disorder patient stabilized from a psychotic standpoint but repeatedly hospitalized due to alcohol-related relapses. The aim is to illustrate the therapeutic and social difficulties encountered when addiction remains active despite psychiatric stabilization.

**Methods:**

A qualitative single case study was conducted of a male patient with chronic psychotic disorder and severe alcohol use disorder. Data were collected through longitudinal clinical observation during several psychiatric hospitalizations and outpatient follow-up. Clinical stability, addictive behaviors, treatment adherence, and social circumstances were analyzed to identify factors contributing to relapse and social exclusion.

**Results:**

The patient remained psychiatrically stable with no delusional or hallucinatory symptoms and displayed good behavior and engagement in care. However, massive alcohol intoxications led to agitated states, neighborhood disturbances, and an ongoing eviction process. Despite partial awareness of his behavior, the patient showed low motivation for addiction treatment. His recurrent hospitalizations were motivated more by social and behavioral issues than by psychiatric decompensation.

**Conclusions:**

This case highlights the clinical and institutional impasse frequently faced in dual disorder management. Psychiatric stabilization alone is insufficient when addiction persists. Coordinated, multidisciplinary strategies involving psychiatric, addiction, and social care are crucial to prevent chronic institutionalization and social rejection.

**Disclosure of Interest:**

None Declared

